# The Bifunctional Pyruvate Decarboxylase/Pyruvate Ferredoxin Oxidoreductase from *Thermococcus guaymasensis*

**DOI:** 10.1155/2014/349379

**Published:** 2014-05-29

**Authors:** Mohammad S. Eram, Erica Oduaran, Kesen Ma

**Affiliations:** ^1^Department of Biology, University of Waterloo, 200 University Avenue West, Waterloo, ON, Canada N2L 3G1; ^2^Structural Genomics Consortium, University of Toronto, Toronto, ON, Canada M5G 1L7; ^3^Department of Chemistry and Physics, Roger Williams University, One Old Ferry Road, Bristol, RI 02809, USA

## Abstract

The hyperthermophilic archaeon *Thermococcus guaymasensis* produces ethanol as a metabolic end product, and an alcohol dehydrogenase (ADH) catalyzing the reduction of acetaldehyde to ethanol has been purified and characterized. However, the enzyme catalyzing the formation of acetaldehyde has not been identified. In this study an enzyme catalyzing the production of acetaldehyde from pyruvate was purified and characterized from *T. guaymasensis* under strictly anaerobic conditions. The enzyme had both pyruvate decarboxylase (PDC) and pyruvate ferredoxin oxidoreductase (POR) activities. It was oxygen sensitive, and the optimal temperatures were 85°C and >95°C for the PDC and POR activities, respectively. The purified enzyme had activities of 3.8 ± 0.22 U mg^−1^ and 20.2 ± 1.8 U mg^−1^, with optimal pH-values of 9.5 and 8.4 for each activity, respectively. Coenzyme A was essential for both activities, although it did not serve as a substrate for the former. Enzyme kinetic parameters were determined separately for each activity. The purified enzyme was a heterotetramer. The sequences of the genes encoding the subunits of the bifunctional PDC/POR were determined. It is predicted that all hyperthermophilic **β**-keto acids ferredoxin oxidoreductases are bifunctional, catalyzing the activities of nonoxidative and oxidative decarboxylation of the corresponding **β**-keto acids.

## 1. Introduction

*Thermococcales* are represented by three genera of* Pyrococcus*,* Thermococcus,* and* Palaeococcus*, which are typically anaerobic chemoorganotrophic microorganisms capable of growing on complex proteinaceous substrates (e.g., yeast extract and tryptone) or carbohydrates (e.g., maltose, starch, and cellobiose). The growth rates of most Thermococcales are stimulated by the addition of elemental sulfur (S^0^) to the growth medium [1].* Thermococcus guaymasensis* is a strictly anaerobic hyperthermophilic archaeon with an optimal growth temperature of 88°C and uses starch, glucose, casein, Trypticase, chitin, dextrose, and maltose as growth substrates to produce acetate, CO_2_, but also propionate, isobutyrate, isovalerate, ethanol, and H_2_ or H_2_S when elemental sulfur is present [2, 3].

Pyruvate is a central metabolic intermediate in most anaerobic hyperthermophilic archaea. The oxidative decarboxylation of pyruvate catalyzed by pyruvate ferredoxin oxidoreductase (POR) leads to the production of acetyl-coenzyme A, which is subsequently converted to acetate as an end product via the enzyme acetyl-coenzyme A synthetase [4–6].* Thermococcales* employ a modified Embden-Meyerhof pathway for glycolysis using various novel enzymes including an ADP-dependent glucokinase and glyceraldehyde-3-phosphate ferredoxin oxidoreductase [7]. Within* Thermococcales*,* P. furiosus *[8],* Thermococcus* strain ES1 [9],* T. guaymasensis* [3], and* Thermococcus onnurineus* [10] are capable of producing ethanol. Different ADHs with potential roles in catalysis of aldehyde reduction and alcohol production are characterized from these organisms [3, 11–13]. An NADP-dependent zinc-containing alcohol dehydrogenase (ADH) with broad substrate range is purified and characterized from* T. guaymasensis* [3]. Similar to several other ADHs characterized from hyperthermophiles, this novel zinc-containing ADH from* T. guaymasensis* possesses low apparent *K*_*m*_ values for aldehydes, suggesting their possible physiological role in the production of alcohols. However, the origin of the aldehydes produced in hyperthermophiles is not well understood [3, 14, 15].

It is known that there are two pathways for ethanol production from pyruvate, and acetaldehyde is an essential intermediate. In a two-step pathway, pyruvate is converted to acetaldehyde by the enzyme pyruvate decarboxylase (PDC, EC 4.1.1.1). In the three-step pathway, pyruvate is transformed into acetyl-CoA that is then reduced to acetaldehyde. ADH is the enzyme that converts acetaldehyde to ethanol. Pyruvate decarboxylase (encoded by* pdc* gene) catalyzes the nonoxidative decarboxylation of pyruvate to acetaldehyde. Within bacteria the enzyme is shown to be present in* Sarcina ventriculi* and* Zymomonas mobilis* [16, 17]. CoA-dependent acetaldehyde dehydrogenase (AcDH, EC 1.2.1.10) is present in few mesophilic and thermophilic prokaryotes (*e.g. Clostridium*,* Thermoanaerobacter*, and* Geobacillus*) and some members of protozoa, such as* Giardia lamblia* and* Entamoeba histolytica*, and catalyzes the conversion of acetyl-CoA to acetaldehyde [18–23].

Since the genome sequence of* T. guaymasensis* is not yet available, it is not possible to determine if the* pdc* gene is present in this organism. A survey of databases containing sequences of hyperthermophilic genomes indicated that none of the hyperthermophilic genome sequences released so far, including the 17 fully sequenced genomes of Thermococcales, bear a gene with homology to any of the well-known acetaldehyde-producing enzymes PDC or AcDH (CoA-acetylating). A bifunctional pyruvate ferredoxin oxidoreductase (POR, EC 1.2.7.1)/pyruvate decarboxylase (PDC) was discovered in the hyperthermophilic archaeon* Pyrococcus furiosus* [6]. The bifunctional enzyme is active in both oxidative and nonoxidative decarboxylation of pyruvate to produce acetyl-CoA and acetaldehyde, respectively. POR seems to be the right candidate to elucidate the unexplained origin of the acetaldehyde in* P. furiosus*.

POR is a ferredoxin-dependent iron-sulfur protein catalyzing the reversible oxidative decarboxylation of pyruvate to acetyl-coenzyme A and CO_2_ with the sequential transfer of the reducing equivalents to ferredoxin, which can be used toward the reduction of the sulfate, N_2_, or protons [24–26]. POR is ubiquitous in archaea and common in bacteria and amitochondriate protists. In aerobic organisms the same reaction is catalyzed via the large enzyme complex pyruvate dehydrogenase (PDH, EC 1.2.4.1). Unlike POR, the reaction catalyzed by PDH is irreversible, NAD^+^ dependent, and uses lipoate and flavin as prosthetic groups [26, 27]. PORs have been purified and characterized from the hyperthermophilic archaea* P. furiosus* [28],* Archaeoglobus fulgidus* [29], and* Sulfolobus sp.* [30, 31]. Within hyperthermophilic bacteria,* Thermotoga maritima* is the only bacterium whose POR has been purified and characterized [32]. It is unclear if the bifunctionality of POR/PDC is only a property of the* Pyrococcus* metabolic system, or it is a general trait of all hyperthermophilic archaea. In this study, the bifunctional PDC/POR enzyme was purified from the hyperthermophilic archaeon* T. guaymasensis*, and its molecular and biochemical properties were characterized. These results will provide a better understanding of metabolic pathway for alcohol production in hyperthermophiles.

## 2. Materials and Methods

### 2.1. Reagents and Chemicals

Sodium pyruvate, thiamine pyrophosphate (TPP), dichloromethane, coenzyme A (CoASH), CAPS (3-(cyclohexylamino)-propanesulfonic acid), EPPS (N-(2-hydroxyethyl)-piperazine-N′-3-propanesulfonic acid), lysozyme, porcine pyruvate dehydrogenase (PDH), and methyl viologen (MV) were purchased from Sigma-Aldrich Canada Ltd. (ON, Canada). Desulfocoenzyme A was synthesized [33]. DNase I for the cell lysis buffer preparation was obtained from Roche (Roche Applied Science, QC, Canada). The chemicals used in the growth media were all commercially available. Yeast extract was acquired from EMD (EMD Chemicals, Inc., NJ, USA) and Trypticase soy broth (TSB) was purchased from Becton-Dickinson (BD Bioscience, Mississauga, ON, Canada). All of the FPLC columns and chromatographic media were purchased from GE Healthcare (QC, Canada). The Hydroxyapatite chromatography material, Bradford reagent, acrylamide, and molecular weight standards were purchased from Bio-Rad Laboratories (ON, Canada).

### 2.2. Growth of Microorganisms

*T. guaymasensis* DSM 11113 and* P. furiosus* DSM 3638 were obtained from DSMZ-Deutsche Sammlung von Mikroorganismen und Zellkulturen (Braunschweig, Germany).* T. guaymasensis* was grown and harvested as described previously [3]. The medium was supplemented with trace minerals and vitamin solutions prepared as previously described by Balch and coworkers [34].* P. furiosus* was grown on maltose, yeast extract, and tryptone at 95°C using the procedures described previously [35]. After centrifugation the cell pellet was frozen in liquid nitrogen and stored at −80°C until use.

### 2.3. Enzyme Purification

To purify the native PORs from* T. guaymasensis* and* P. furiosus*, cell-free extracts (CFEs) were prepared from the cells grown to late log-phase. All purification steps were performed under strictly anaerobic conditions and at room temperature, unless otherwise specified.

Cell pellets stored at −80°C were resuspended in degassed lysis buffer 50 mM Tris-HCl pH 7.8, 5% glycerol, 2 mM dithiothreitol (DTT), 2 mM sodium dithionite (SDT), 0.1 mg mL^−1^ lysozyme, and 0.01 mg mL^−1^ DNaseI in a sealed and degassed flask. The ratio of cells (wet weight) to lysis buffer was 1 : 6 (w/v) for* T. guaymasensis* and 1 : 4 (w/v) for* P. furiosus*. The cell suspensions were incubated at 37°C while stirring for 2 h and subsequently centrifuged anaerobically at 10,000 ×g for 30 min at 4°C. The supernatant, designated as cell-free extract (CFE), was transferred to an anaerobic serum bottle using a syringe prerinsed with anaerobic buffer A (50 mM Tris-HCl pH 7.8, 5% glycerol, 2 mM DTT, and 2 mM SDT) and used directly as the starting materials for purification.

All purification steps were performed using an FPLC system (GE Healthcare, QC, Canada). The PDC and POR activities were monitored during the chromatography by enzyme assays to confirm coelution of the activities. At each step the purity of the active fractions was determined by SDS-PAGE.

The CFE of* T. guaymasensis* was applied to a diethylaminoethyl- (DEAE-) Sepharose column (5.0 × 10 cm) equilibrated with anaerobic buffer A. The column was washed with 3 column volumes (CVs) of buffer A and then a linear gradient using buffer A and 50% of buffer B 50 mM Tris-HCl pH 7.8, 5% glycerol, 1 M sodium chloride, 2 mM DTT, and 2 mM SDT applied at a flow rate of 8.0 mL min^−1^. The fractions (160–220 mM NaCl) with enzymatic activities were pooled and loaded on a hydroxyapatite column (HAP, 2.6 × 15 cm) equilibrated with buffer A. The column was washed with 100 mL of buffer A, and then the absorbed proteins were eluted with a linear gradient (0–0.5 M phosphate) using buffer A and 100% buffer C (50 mM Tris-HCl pH 7.8, 5% glycerol, 0.32 M K_2_HPO_4_, 0.18 M KH_2_PO_4_, 2 mM DTT, and 2 mM SDT) at a flow rate of 2.5 mL min^−1^ and 10 CVs were applied. The fractions (110–145 mM potassium phosphate) with enzymatic activities were pooled and mixed with 40% (v/v) of buffer D (50 mM Tris-HCl pH 7.8, 5% glycerol, 2 M ammonium sulfate, 2 mM DTT, and 2 mM SDT) before loading onto a Phenyl-Sepharose column (PS, 2.6 × 10 cm) equilibrated with buffer A containing 0.8 M ammonium sulfate. After loading, the column was washed with two CVs of buffer A containing 40% of buffer D, and then a decreasing linear gradient from 40% to 0% buffer D in buffer A and 8 CVs was applied at a flow rate of 2.5 mL min^−1^. The POR was eluted at 440–290 mM ammonium sulfate, and the purity was confirmed by SDS-PAGE. The purified fractions were then desalted and concentrated under anaerobic conditions using an ultrafiltration device (Advantec MFS, Inc., CA, USA) with a 44.5 mm membrane of polyethersulfone and nominal molecular weight limit (NMWL) of 50,000 (Millipore, MA, USA) under N_2_. After concentration, the purified POR from* T. guaymasensis* (TgPOR) in buffer A was stored as small droplet in liquid nitrogen until use.

To purify the bifunctional PDC/POR from* P. furiosus* (PfPDC/POR) a modification of the procedure described by Blamey and Adams [28] was used. The CFE was loaded onto a DEAE-Sepharose column (5.0 × 10 cm) equilibrated with anaerobic buffer A and was washed with 2.5 CVs of buffer A followed by a linear gradient from 0 to 50% buffer B in buffer A at 6 CVs at a flow rate of 5.0 mL min^−1^. The active fractions (162–198 mM NaCl) were pooled and mixed with 50% of buffer D before loading onto a Phenyl-Sepharose column (2.6 × 10 cm) equilibrated with buffer A containing 1.0 M ammonium sulfate. The column was then washed with 1.5 CVs of the buffer A containing 50% (v/v) buffer D, and a linear decreasing gradient from 50% to 0% buffer D in buffer A at 12 CVs was applied at a flow rate of 3.0 mL min^−1^. The PfPDC/POR was eluted from the Phenyl-Sepharose column at 900–700 mM ammonium sulfate. The active fractions were pooled, desalted, and concentrated using the ultrafiltration system as described for TgPOR/PDC. The concentrated fraction was then loaded onto a HiLoad Superdex-200 (GE healthcare, QU, Canada) gel-filtration chromatography column (2.6 × 60 cm) equilibrated with buffer E (50 mM Tris pH 7.8, 5% glycerol, 100 mM KCl, 2 mM DTT, and 2 mM SDT) and eluted at the flow rate of 2 mL min^−1^. The active fractions were then pooled and loaded onto a hydroxyapatite column (2.6 × 15 cm) equilibrated with buffer A at 4.0 mL min^−1^. The column was then washed with 100 mL of buffer A followed by elution using an increasing linear gradient from 0 to 0.5 M phosphate (buffer A to 100% buffer C) at 4 CVs. The purified PfPDC/POR, as judged by SDS-PAGE, was eluted from the HAP at 90–140 mM phosphate. The purified PfPDC/POR was desalted, concentrated, and stored in liquid nitrogen until use.

### 2.4. Enzyme Assays

Pyruvate decarboxylase activity (PDC) was determined by measuring the rate of acetaldehyde production as described previously [6] with some modifications. In principle, acetaldehyde produced during the enzymatic reaction was derivatized with an acidic solution of 2, 4-dinitrophenylhydrazine (DNPH). The reaction with aldehyde groups formed a yellow-reddish color resulting from the formation of the corresponding hydrazone derivative, which was quantified by reverse-phase high performance liquid chromatography (RP-HPLC).

The reactions were carried out in stopper-sealed 8 mL vials under anaerobic conditions at 80°C unless otherwise specified. The assay mixture (1 mL final volume) contained EPPS buffer (100 mM, pH 8.4), 1 mM MgCl_2_, 0.1 mM TPP, 10 mM sodium pyruvate, and 1 mM CoASH. Unless specified, the enzyme reactions were stopped after 20 min by transferring the assay vials to ice and adding 2 mL of saturated DNPH solution in 2 N HCl. The vials were then incubated overnight at room temperature with shaking (150–200 rpm) to allow derivatization of acetaldehyde. The resulting hydrazone derivative was then extracted twice with 1 mL of dichloromethane (DCM) with vigorous shaking at room temperature for 15 min. The organic (lower) phase was then transferred to a clean vial and placed in a vacuum desiccator connected to a water pump to evaporate the DCM. After evaporation of DCM (about 3-4 h), the resulting yellowish-red powder was dissolved in 4 mL of acetonitrile (HPLC grade) by overnight incubation at 4°C.

An aliquot of the reaction product was filtered through a 0.2 *μ*m nylon syringe filter (National Scientific, Rockwood, TN, USA) and analyzed using a Perkin-Elmer LC series 4 HPLC system (Norwalk, CT, USA) fitted with a reversed-phase Allure C18 column (150 × 4.6 mm, 5 *μ*m, 60 Å). Isocratic elution conditions with a mobile phase of acetonitrile/water (80 : 20 v/v) were used at a flow rate of 1 mL min^−1^. The reaction product was detected with a micrometrics model 788 dual variable wavelength detector (Norcross, GA, USA) at 365 nm. The sample was applied using a Rheodyne Model 7125 injection valve (Rheodyne Inc., CA, USA) with a 20 *μ*L sample loop. The HPLC system was operated at room temperature. The final concentration of acetaldehyde was determined using a standard curve from known concentrations of acetaldehyde processed under the same assay conditions.

The pyruvate- and coenzyme A-dependent reduction of the methyl viologen was measured for POR activities under strictly anaerobic conditions [28]. The assays were carried out at 80°C in an assay mixture (2 mL) containing 100 mM EPPS, pH 8.4, 1 mM MgCl_2_, 5 mM sodium pyruvate, 0.4 mM TPP, 100 *μ*M CoASH, 1 mM of methyl viologen (MV), and enzyme in stoppered optical glass cuvettes with 1 cm light path (Starna cells, Inc., Atascadero, CA, USA). In addition, a small amount of SDT was added to the assay mixture (until a light blue color appeared) to scavenge residual oxygen and slightly reduce the assay mixture before the addition of the enzyme. The absorbance changes at 578 nm were monitored. An extinction coefficient of *ε*_578_ = 9.8 mM^−1^ cm^−1^ [36] was used for calculating activity. The oxidation of one pyruvate would release two electrons. The activity was calculated based on the initial linear part of enzymatic reaction progress curve, and one unit of enzyme activity was defined as the oxidation of 1 *μ*mol of the substrate or the reduction of 2 *μ*mol MV per minute. A linear correlation between the activity and the amount of protein in the assay was confirmed.

To determine if pyruvate dehydrogenase (PDH) has the ability to catalyze the production of acetaldehyde from pyruvate, a modified PDC assay was used. The PDC/POR enzyme was replaced with the porcine PDH, and the reaction was performed at 30°C. The assays were carried out under aerobic conditions and 0.1 M buffer at pH values of 6.2 and 7.5 (sodium phosphate), 8.4 (EPPS), and 10.2 (CAPS). The standard assay mixture (1 mL final volume) contained 1 mM MgCl_2_, 2.4 mM NAD^+^, 0.5 mM TPP, 5 mM sodium pyruvate, and 0.5 mM CoASH with or without 0.3 mM DTT and was preincubated in water bath (30°C) for 4 min. The reactions were started by enzyme addition and ended after 90 min. Control assays were performed, which included reactions with no enzyme, no CoA, and no pyruvate.

CoA-dependent acetaldehyde dehydrogenase activity was assayed using CFEs of* T. guaymasensis* and* P. furiosus*. The assays were carried out at 80°C in an assay mixture (2 mL) composed of buffer (sodium phosphate, pH 6.0, EPPS, pH 8.4, or glycine, at pH 10.5 all at 50 mM), 1 mM MgCl_2_, 1 mM DTT, 0.1 mM CoASH, and 1.5 mM NAD^+^ or NADP^+^. The anaerobic assay buffer was transferred to a degassed assay cuvette using syringes prerinsed (three times) with anaerobic buffer. The cuvette was incubated in a water-jacketed cuvette holder on a Genesys 10 UV-Vis spectrophotometer (Thermo Scientific, MA, USA) and preincubated to the assay temperature (80°C) for 4 min. Assay components were added using prerinsed Hamilton gas-tight syringe (Hamilton Company, Reno, NV, USA) in rapid succession, and the assay mixture in the cuvette was further incubated for another 30 s. The reaction was started by adding acetaldehyde. The absorbance change at 340 nm was monitored.

### 2.5. Biochemical and Biophysical Characterization

To determine the pH dependence of each activity, assays were carried out, at different pH values ranging from 6.0 to 11 using TgPDC/POR. All of the pH values were adjusted and measured at room temperature. The buffers used were sodium phosphate buffer (Δp*K*_*a*_/°C = −0.0028) for pH values of 6.0, 7.0, and 7.5, EPPS buffer (ΔP*K*_*a*_/°C = −0.015) for pH values of 7.5, 8.0, and 8.4, glycine buffer (Δp*K*_*a*_/°C = −0.0025) for pH values of 8.5, 9.0, 9.5, 10.0, and 10.5, and finally CAPS buffer (Δp*K*_*a*_/°C = −0.009) for pH values of 10.0, 10.5, and 11.0.

Enzyme kinetics parameters (*K*_*m*_ and *V*_max⁡_) were determined for each activity at the optimal pH and under strictly anaerobic conditions. All of the kinetic experiments were carried out at 80°C. The apparent kinetic parameters were determined for pyruvate, TPP, coenzyme A, and the artificial electron acceptor methyl viologen (MV) by applying various concentrations of each component and keeping the concentration of other assay components invariable. The kinetic parameters were calculated from best fit of data to the Michaelis-Menten equation using SigmaPlot software (SYSTAT Software Inc., CA, USA).

To investigate oxygen sensitivity of each activity, enzyme in buffer A was exposed to air at 4°C by gentle stirring. Enzyme activities were then measured at different time points and compared to a control preparation that was kept on ice and under anaerobic conditions (unexposed control).

The temperature dependencies of both activities were determined by assaying the enzyme activity at different temperatures from 30°C to 95°C under standard assay conditions. The POR assays were carried out in 100 mM EPPS buffer containing 10 mM MgCl_2_, at pH 8.4. To determine the half-life of the enzyme in buffer A at 80°C, the residual activities at different time points were determined and compared to the unheated control.

### 2.6. Sequencing of* por*/*vor* Operon

Since no genome sequence of* T. guaymasensis* is available, the genes encoding the POR and the closely related enzyme VOR were sequenced using a primer walking strategy. Primers were designed based on highly conserved regions (including CoA-binding, TPP-binding, and [4Fe-4S] cluster-binding motifs) within* por*/*vor* operons of coding sequences of the* Thermococcales* genomes including* T. kodakaraensis*,* P. furiosus*,* Pyrococcus horikoshii*, and* Pyrococcus abyssi*. In some cases degenerated primers were designed and used for PCR. The PCR products with sizes close to the expected PCR product (estimated based on the closely related species) were sequenced. The identity of newly sequenced stretches of DNA was confirmed by homology searching in the databases. The sequenced fragments were then used to design next set of primers for amplification of the new fragments of genomic DNA. In some cases, when the primer walking strategy failed to produce a positive PCR product, inverse PCR (IPCR) was used to obtain the neighboring sequences.

### 2.7. Other Methods

The protein concentration was routinely determined using Bradford dye-binding method [37]. Bovine serum albumin (BSA) was the standard. SDS-PAGE was used to monitor the purity of the enzymes and subunit Mrs [38].

The native molecular weight of TgPDC/POR was estimated by loading the concentrated protein (approximately 1 mg) on a HiLoad Superdex-200 size-exclusion chromatography column of (2.6 × 60 cm, GE Healthcare, QC, Canada) equilibrated with buffer E at the flow rate of 2.5 mL min^−1^. The following standards from the Pharmacia protein standard kit (Pharmacia, NJ, USA) were applied to the column: blue dextran (2,000,000 Da), thyroglobulin (669,000 Da), ferritin (440,000 Da), catalase (232,000 Da), aldolase (158,000 Da), bovine serum albumin (67,000 Da), ovalbumin (43,000 Da), chymotrypsinogen A (25,000 Da) and ribonuclease A (13,700 Da).

## 3. Results

### 3.1. Enzyme Purification

Both* T. guaymasensis* and* P. furiosus* were grown to late log or early stationary phase, and the cells were harvested by continuous centrifugation. The PDC and POR activities were determined to be 0.04 ± 0.01 U mg^−1^ and 2.6 ± 0.2 U mg^−1^ in CFE of* T. guaymasensis*, respectively. The activities in CFE of* P. furiosus* were 0.03 ± 0.011 and 3.2 ± 0.12 U mg^−1^, respectively.

**Figure 1:**
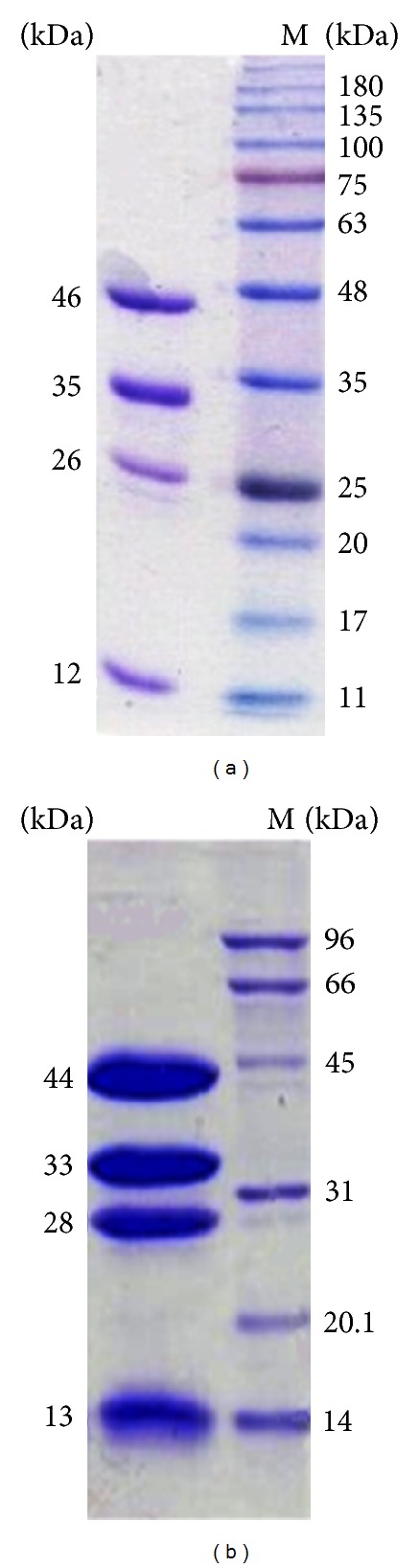
SDS-PAGE analysis of purified enzymes. (a) 5 *μ*g of the purified TgPDC/POR; lane M, BLUeye prestained protein ladder. (b) 12 *μ*g of the purified PfPDC/POR; lane M, molecular weight standard markers. The numbers on the right side indicate the molecular weight of the marker bands and the numbers on the left side indicate the estimated molecular weights on the subunits.

PDC and POR activities coeluted from all columns during purification (data not shown). Representative purifications for the enzymes from* T. guaymasensis* (Table 1) and* P. furiosus* (Table 2) resulted in a relatively low recovery (about 7-8%), but their purity was confirmed by SDS-PAGE analysis (Figures 1(a) and 1(b)). The purified proteins from both organisms had a brownish color, which is a hallmark of iron-sulfur proteins. The purified TgPDC/POR had specific activities of 3.8 ± 0.22 mg^−1^ and 20.2 ± 1.8 U mg^−1^ for PDC and POR, respectively. The purified PfPDC/POR had specific activities of 4.3 ± 0.3 U mg^−1^ and 22.3 ± 2.2 U mg^−1^ for PDC and POR, respectively. Purification of PfPOR resulted in a specific activity increase of 7.8-fold over that of CFE, which was in agreement with the previous report [28]. The increase in specific activities for both PDC and POR from CFE to purified enzyme appeared not to be proportional, and the PDC specific activity increased much more greatly. This might have resulted from two possible factors. The POR may have been overestimated in CFE due to the presence of VOR, which can also catalyze the oxidative decarboxylation of pyruvate [39, 40]. Secondly, the PDC activity in CFE may have been underestimated due to the low sensitivity for acetaldehyde at low concentrations.

The extensive database search to find a gene or protein homolog of the CoA-dependent AcDH in hyperthermophilic archaea and bacteria failed. The available genome sequences from different archaeal and bacterial hyperthermophiles were searched based on both the annotations and the homology to the known* adhE *(e.g., those from* Lactobacillus acidophilus* (accession number YP_193379) and* E. coli* (accession number NP_415757)) and* mhpF* (e.g. those of* E. coli* (accession number YP_488645) and* Bacillus megaterium* (accession number YP_003564035)) sequences. Each genome of hyperthermophilic archaea and bacteria was separately searched for annotations, and BLAST searches were performed for homologs of the each type of the CoA-dependent AcDH. In addition, CoA-dependent acetaldehyde dehydrogenase activity under the conditions examined (different pHs and buffers, different amounts of CFEs, and using NAD^+^ and NADP^+^ as cofactors) was not detectable in CFEs of two hyperthermophilic archaea,* T. guaymasensis* and* P. furiosus*.

### 3.2. Biophysical and Catalytic Properties

The apparent Mr of the purified TgPDC/POR was determined to be 258, 000 ± 5,600 (*n* = 3) by gel-filtration chromatography column. The SDS-PAGE analysis of the purified enzyme after gel-filtration column (Figure 1(a)) revealed that the enzyme was composed of four different subunits, with estimated molecular weights of 12,000, 26,000, 35,000, and 46,000. These weights were further verified by in silico translation of the nucleotide sequence of each subunit, yielding weights of 11.9, 19.9, 36.1, and 43.8 kDa. Overall, these results indicated that the native molecule was a dimer of heterotetramers (*α*_2_ *β*_2_ *γ*_2_ *δ*_2_), which was very similar to that of PfPDC/POR.

**Figure 2:**
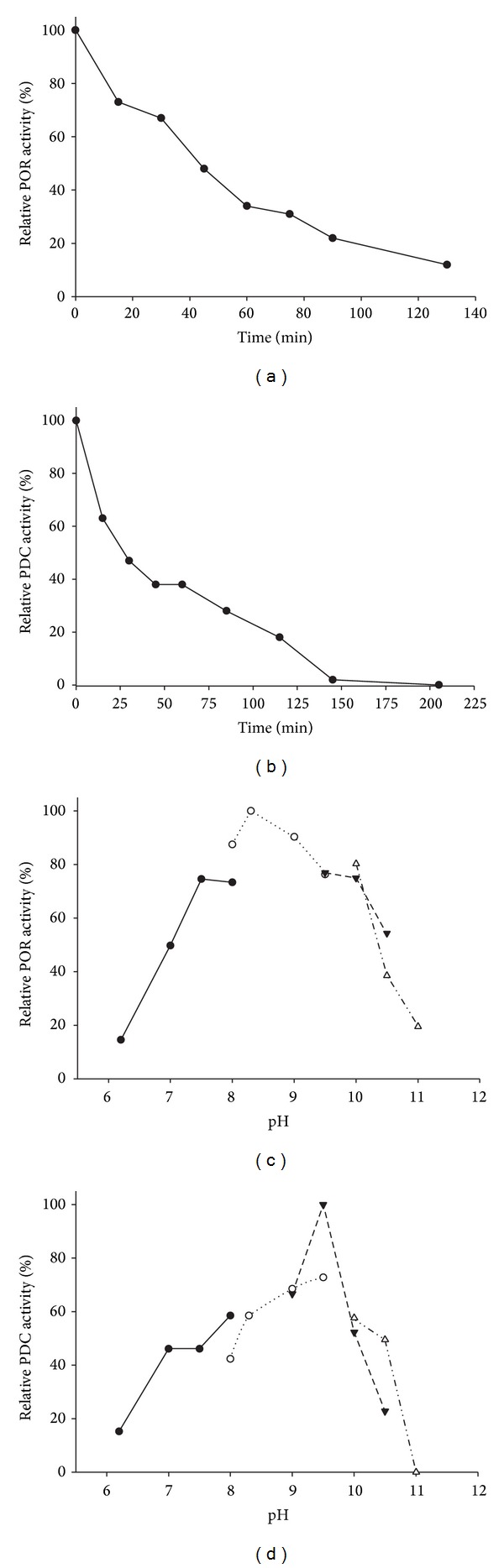
Oxygen sensitivity and optimal pH for* T. guaymasensis* PDC and POR activities. The effect of air exposure on (a) POR and (b) PDC reactions was measured at different time points in TgPOR/PDC aliquots exposed to air while stirring. The relative activities of 100% equal the highest measured specific activity at time zero with no exposure to air (16.2 U mg^−1^ and 2.2 U mg^−1^ for POR and PDC activities, resp.). The effect of pH on (c) POR and (b) PDC activities of TgPOR/PDC was determined under strictly anaerobic conditions at 80°C. The relative activities of 100% equal the highest measured specific activities (17.4 U mg^−1^ and 2.6 U mg^−1^ for POR and PDC, resp.). For the optimal pH determination experiments the filled circles represent the reactions with sodium phosphate buffers (pH 6.2, 7.0, 7.5, and 8.0), the open circles represent the reactions with EPPS buffer (pH 8.0, 8.4, 9.0, and 9.5), the filled triangles represent glycine buffer (pH 9.5 and 10), and open triangles represent the CAPS buffer (pH 10.5 and 11.0).

Both POR and PDC activities were oxygen sensitive. The time required for a 50% loss of POR activity (*t*_1/2_) of the purified enzyme (1.3 mg mL^−1^ in 50 mM Tris-HCl (pH 7.8) containing 2 mM SDT and 2 mM DTT) was about 40 min at 4°C (Figure 2(a)). The *t*_1/2_ for the PDC activity (0.8 mg mL^−1^ in 50 mM Tris-HCl (pH 7.8) containing 2 mM SDT and 2 mM DTT) was approximately 30 min (Figure 2(b)). Ideally, the sensitivity of both activities should have been determined on the same batch of the enzyme; however, due to technical difficulties including different assay procedures, this was not possible. Therefore, the different *t*_1/2_ might simply reflect experimental differences.

The effect of pH on both POR and PDC activities was determined. TgPOR showed significant activity (more than 75% of maximum) in a relatively wide pH range from 7.5 to 10, with the highest at pH 8.4 (Figure 2(c)). The optimal pH for PDC activity displayed a relatively sharp optimum at pH 9.5 (Figure 2(d)).

**Figure 3:**
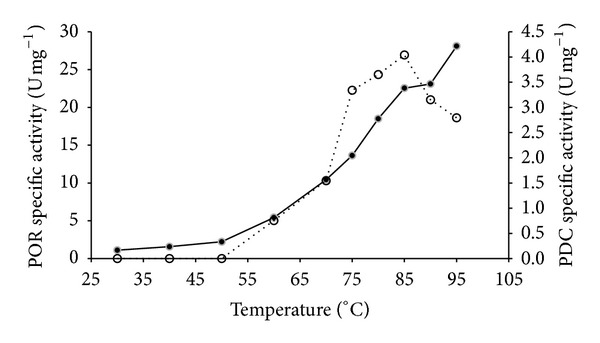
Temperature dependence of* T. guaymasensis* PDC and POR activities. The open circles represent the PDC and the filled circles represent the POR activity.

The optimal temperatures for both POR and PDC activities were determined. The reaction rates of both POR and PDC increased along with the increasing assay temperature. The optimal temperature for POR activity was above 95°C (Figure 3). In the case of the PDC activity, the reaction rate increased up to 85°C. PDC activity decreased at assay temperatures above 85°C (Figure 3).

Enzyme kinetic parameters were obtained from the nonlinear regression of Michaelis-Menten plots for enzymes from* T. guaymasensis* and* P. furiosus* (Table 3). The bifunctional PfPOR/PDC was used for the purpose of control or comparison. Both activities of POR and PDC displayed a typical Michaelis-Menten progress curve for pyruvate and CoA.

Both TgPDC and TgPOR activities were absolutely dependent on CoA, and no activity was observed when it was omitted from the assay mixture. No other components (ATP, ADP, pantothenic acid, and various combinations of them) could substitute CoA (data not shown). It was observed that desulfo-CoA, an analogue of CoA, was completely inhibitory for the oxidation (POR) reaction, but not for the decarboxylation (PDC) reaction. When desulfo-CoA was substituted for CoA in the PDC assay, the enzymes from* T. guaymasensis* and* P. furiosus* exhibited approximately 75% and 80% of the activities with CoA, respectively. The ability of the PDC activity to utilize desulfo-CoA instead of CoA supported the previously suggested structural rather than catalytic role of CoA in the PDC reaction [6].

The results of the PDC assay on the commercially purchased porcine PDH indicated that this enzyme was unable to catalyze the production of acetaldehyde from pyruvate. There were some residual amounts of acetaldehyde detected in each vial; however, this residual activity was lower than the amount detected in the negative controls, which contained no enzyme and/or no CoA (data not shown). This trace amount of acetaldehyde can be attributed to the nonenzymatic decarboxylation of pyruvate in the presence of reducing agents, which was reported previously [41].

### 3.3. Sequencing and Phylogenetic Analyses of* por(pdc)*/*vor*

**Figure 4:**
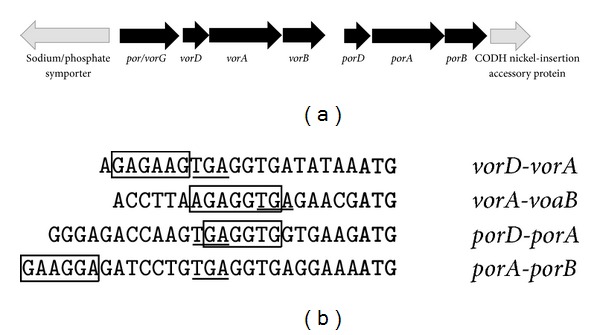
Sequence analysis of the* T. guaymasensis*'s* por(pdc)*/*vor* operon. (a) Gene organization of* por(pdc)*/*vor* gene cluster in* T. guaymasensis*. (b) Intercistronic sequences in* por(pdc)*/*vor* operon of* T. guaymasensis*. The translation initiation (start) codons of the distal genes are indicated in bold, the ribosome-binding site (RBS) of the distal gene is boxed, and the translation terminations of the proximal genes are underlined.* por*, pyruvate ferredoxin oxidoreductase;* vor*, 2-ketoisovalerate ferredoxin oxidoreductase; CODH, carbon monoxide dehydrogenase.

A total of 6.7 kbp of the genome of* T. guaymasensis* was sequenced using primer walking and inverse PCR strategies and deposited in the EMBL/GenBank/DDBJ nucleotide sequence databases under the Accession No. KC262637. The sequences obtained encompassed genes encoding all of the subunits of PDC/POR and VOR. Parts of the neighboring sequences were also retrieved. By analysis of the sequence information from the* por(pdc)*/*vor* gene cluster in* T. guaymasensis* and comparison with other hyperthermophilic* por*/*vor* sequences from different databases, the gene organization in* T. guaymasensis* was determined (Figure 4(a)). The deduced nucleotide sequences encoded for proteins with 186, 105, 391, 311, 105, 394, and 332 amino acid residues of Por/VorG, VorD, VorA, VorB, PorD, PorA, and PorB, respectively.

The gamma subunit Por/VorG is shared between POR and VOR, which is a common property of the* por*-*vor* operon in* Thermococcales* [42]. The subunits* vorD*,* vorA*, and* vorB* of VOR were located downstream of the* por*/*vorG*, and then the* porA* and* porB* were located downstream of the* vor* genes.

**Figure 5:**
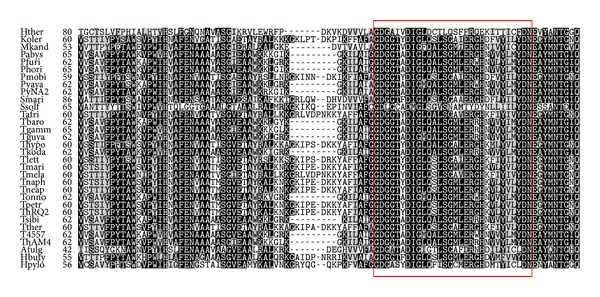
Alignment of part of PorB including the TPP-binding motif from various bacteria and archaea. Amino acid sequences of PorB were retrieved from GenBank and aligned using ClustalW. Alignments were colored using Boxshade 3.21 (EMBnet.org). The TPP-binding motif (GDGX^24–27^NN) is boxed. The abbreviations and accession numbers are as follows: Afulg,* Archaeoglobus fulgidus* (YP_007906346); Hpylo,* Helicobacter pylori* (YP_627791), Hbuty,* Hyperthermus butylicus* (YP_005260918); Koler,* Kosmotoga olearia* (YP_002940111); Mkand,* Methanopyrus kandleri* (NP_613370); Pmobi,* Petrotoga mobilis* (YP_001567641); Pabys,* Pyrococcus abyssi* (NP_127037); Pfuri,* Pyrococcus furiosus* (NP_578694); Phori,* Pyrococcus horikoshii* (NP_142634); PyNA2,* Pyrococcus* species strain NA2 (YP_004424217); Pyaya,* Pyrococcus yayanosii* (YP_004623620); Smari,* Staphylothermus marinus* (YP_001041448); Ssolf,* Sulfolobus solfataricus* (BAB70484); Tafri,* Thermosipho africanus* (YP_002334348); Tmela,* Thermosipho melanesiensis* (YP_001305598); ThAM4,* Thermococcus* species strain AM4 (YP_002581949); Tbaro,* Thermococcus barophilus* (YP_004071752); Tnaph,* Thermotoga naphthophila* (YP_002534222); Tlett,* Thermotoga lettingae* (YP_001471392); Tneap,* Thermotoga neapolitana* (YP_002534222); Thypo,* Thermotoga hypogea* (AGO81713); Tther,* Thermotoga thermarum* (YP_004660965); Tmari,* Thermotoga maritima* (NP_227834); ThQ2,* Thermotoga* species strain RQ2 (NA);T4557,* Thermococcus* species strain 4557 (YP_004763195); Tsibi,* Thermococcus sibiricus* (YP_002994778); Tgamm,* Thermococcus gammatolerans* (YP_002958627); Tkoda,* Thermococcus kodakaraensis* (YP_184397).

The TgPOR subunits PorA, PorB, and PorD contained 1, 6, and 8 conserved cysteine residues, respectively. Unlike PORs of* Thermotogales*, which contained two highly conserved cysteine residues per PorG subunit, there were no cysteines present in* Thermococcales PorD*. The primary sequence analysis confirmed the presence of two typical highly conserved cysteine-rich ferredoxin type [4Fe-4S] cluster binding motifs (CXXCXXCXXXCP) in PorD. There were four highly conserved cysteine residues in the PorB, which were believed to be involved in the coordination of the third iron-sulfur cluster for the electron transfer to low molecular weight ferredoxin molecules [42]. Additionally two other cysteine residues were found to be conserved in all* Thermococcales* (except for* Thermococcus sibiricus* and* Thermococcus barophilus*). The PorB also contained the conserved TPP-binding motif (Figure 5), which is the common feature of all TPP-dependent enzymes and is known to be involved in Mg^2+^-TPP cofactor binding [43, 44].

## 4. Discussion

Many hyperthermophiles can grow on peptides and/or sugars via a fermentative-type metabolism. As expected, several alcohol dehydrogenases have been found in different ethanologenic hyper/thermophiles, including,* Thermotoga hypogea* [14],* Thermococcus* strain ES1 [45],* T. guaymasensis* [3],* T. onnurineus* [10],* T. kodakaraensis* [46], and* Carboxydothermus hydrogenoformans* [47]. However, the absence of the commonly known pyruvate decarboxylase (PDC) is a perplexing feature of hyperthermophiles [15]. PDC is widely distributed in plants and fungi but has very limited distribution in bacteria and so far has not been detected in animals [48]. The only PDC activity that has been reported from hyperthermophiles is a bifunctional POR/PDC isolated from the heterotrophic hyperthermophilic archaeon* P. furiosus* [6, 9]. The current study provided evidence confirming that POR of* T. guaymasensis* is also able to catalyze the nonoxidative decarboxylation of pyruvate to produce acetaldehyde.

The purification scheme of TgPOR (Table 1) was similar to previously published report [28] on purification of the similar enzyme from the closely related archaeon* P. furiosus* (Table 2). The purified enzyme from* T. guaymasensis* eluted as a single peak from a Superdex-200 gel-filtration column, indicating the purity of the protein preparation. On SDS-PAGE, four subunits were revealed (Figure 1(a)) which is in accordance with sequencing information acquired (Figure 4(a)), and the subunit composition of similar enzymes from various organisms. Both enzymes purified from* T. guaymasensis* and* P. furiosus* were heterotetrameric proteins, with native molecular weights of approximately 260 kDa, which was indicative of the native protein being a dimer of tetramers (*α*_2_ *β*_2_ *γ*_2_ *δ*_2_ structure).

Like other archaeal PORs characterized so far, including* P. furiosus* [28] and* A. fulgidus* [29], the addition of TPP to the standard assay mixture had no effect on the rate of pyruvate oxidation or decarboxylation when the enzymes were assayed in the crude cell extracts or CFEs of* T. guaymasensis*. Additional TPP also had no impact on the PDC activity of the bifunctional enzyme from* P. furiosus* ([6] and this study). However, incorporation of TPP to the assay mixtures led to a 20–25% stimulation of both POR and PDC activities catalyzed by the purified enzyme from* T. guaymasensis*. This was presumably due to a partial dissociation of the TPP from the enzyme during purification, which is a common feature of bacterial PORs but exceptional within archaeal PORs. Because the enzyme exhibited a significant portion of its POR and PDC activities in the absence of additional TPP, the apparent kinetic parameters for TPP were not determined.

Both TgPDC and TgPOR activities catalyzed by the purified enzyme were highly oxygen sensitive, which is a typical characteristic of the majority of PORs isolated and characterized and different from the commonly known PDCs. Upon exposure to air, the bifunctional TgPDC/POR lost 50% of its POR and PDC activities within 40 and 30 min, respectively.

The TgPOR activity displayed an optimum at pH 8.4, which is in accordance with the previous findings on the corresponding enzyme from closely related archaeon* P. furiosus* [28] as well as the hyperthermophilic bacterium* T. maritima *[32]. However, TgPDC activity displayed an optimal pH of 9.5, which is close to the value reported for the similar bifunctional enzyme from* P. furiosus *[28]. The physiological significances of these differences are not clear. The pH optima for both archaeal bifunctional PDCs were higher than those of the commonly known bacterial and fungal PDCs, which function more efficiently at slightly acidic to neutral (between 5.0 and 7.5) pH ranges [16, 17, 49–51].

Optimal temperature for TgPOR activity was determined to be above 95°C, which is similar to those of* P. furiosus* [28] and* T. maritma* [32], which have growth temperature optima of about 100°C and 80°C, respectively. However, TgPDC activity of the purified enzyme had an optimal temperature of 85°C. As in many other studies, the temperature ranges higher than 95°C were not investigated due to instability of the assay components at such high temperatures as well as the technical difficulties of conducting assays at such higher temperatures.

Differences in pHs, optimal temperatures, and *K*_*m*_ and *V*_max⁡_ values for TgPDC and TgPOR may indicate significant differences in catalytic centers responsible for each of the activities. Since there is no knowledge about the existence of a second coenzyme A-binding site in PORs, it would be plausible to assume that the change in structural microenvironment must occur upon the binding of coenzyme A, which would enable the catalysis of nonoxidative decarboxylation of pyruvate. However, further investigation is required, and probably an X-ray crystallography study would provide helpful information for understanding the differences in catalytic mechanisms.

Nucleotide sequence of* por(pdc)*/*vor* operon in* T. guaymasensis* indicated that POR and VOR were each encoded by four genes, with one of them (*por*/*vorG*) shared between the two enzymes. This is in accordance with the gene organization and enzyme structure of other archaeal hyperthermophiles studied to date [26, 42]. Bacterial hyperthermophiles, including* T. hypogea* and* T. maritima*, do not possess the VOR, and only the four subunits of POR are found (Eram & Ma, unpublished data). As expected, the amino acid sequences of TgPOR subunits were remarkably similar to the corresponding subunits of other *β*-keto acid oxidoreductases, especially to PORs from various hyperthermophiles. The shared subunit (Por/VorG) seems to be expressed independently of each of POR and VOR, and it might be suggestive that the enzymes are not necessarily being expressed simultaneously.

Beta-keto acid oxidoreductases (KORs) including POR, VOR, *β*-ketoglutarate oxidoreductase (KGOR) and indolepyruvate oxidoreductase (IOR) play central roles in amino acid degradation pathways in the majority of sulfur-dependent hyperthermophilic archaea [1]. Other than POR, which is involved in both sugar and amino acid metabolism, other enzymes of the KOR family are exclusively found in hyper/thermophilic heterotrophic and methanogenic archaea [39, 40, 52, 53]. When grown on peptides, *β*-keto acids produced from transamination of different amino acids are oxidized by the corresponding KORs. The *β*-ketoglutarate (KGOR), aromatic aryl acids (IOR), the keto acids derived from the branched chain amino acids (VOR), and pyruvate (POR) are oxidized to the corresponding aryl- or acyl-CoA compounds, with concomitant release of CO_2_. The final step in archaea is catalyzed by acetyl-coenzyme A synthetase I and II (ACS), which results in coupling energy conservation in the form of ATP [1, 53]. Generally, KORs do not have a broad substrate range and act very specifically, although some levels of overlapping substrate specificity exist [39, 40].

The microarray studies show a constitutive transcription of KORs, including the* por* and* vor*, when* P. furiosus *is grown on peptides [54]. Even whenthe organism is cultivated on maltose, the transcription of the genes encoding four POR subunits displays no major changes (unlike other KOR genes, which show decreased transcription), which is most likely due to the role of POR in the metabolism of pyruvate produced from glycolysis [54]. This is also in accordance with the gene organization of the* por*/*vor* operon determined in* T. guaymasensis*, in which three transcription units were identified: the* por*/*vorG*,* vorDAB*, and* porDAB*. Then for expression of each enzyme (POR or VOR), two of the transcription units would be transcribed and eventually translated. Transcription levels of the POR subunit encoding genes are not affected during the early (1-2 h) and late (5 h) cold-shock (72°C) response, which is consistent with no change in POR activity [55].

**Figure 6:**
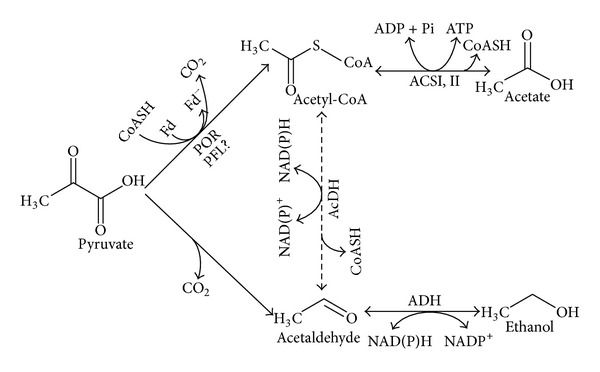
Proposed pathway of ethanol production in* T. guaymasensis*. POR, pyruvate ferredoxin oxidoreductase; PDC, pyruvate decarboxylase; ADH, alcohol dehydrogenase; ACS, acetyl-CoA synthetase; AcDH, acetaldehyde dehydrogenase. The solid arrows indicate the steps confirmed by enzyme assays, while dashed arrow indicates possible pathway that has not been proved.

The ADH isolated and characterized from* T. guaymasensis* is suggested to be involved in the NADP^+^ regeneration by concomitant production of acetoin and ethanol [3]. The proposed pathway for producing ethanol in* T. guaymasensis* is presented in Figure 6. There are two possible pathways for the production of acetaldehyde, which are (a) the consecutive reactions of POR and/or pyruvate formate lyase (PFL) followed by AcDH (CoA-dependent) and (b) the direct production of acetaldehyde from pyruvate by the enzyme PDC (Figure 6), though the former is unlikely as a CoA-dependent acetaldehyde dehydrogenase activity was not detected.

The genes encoding CoA-dependent acetaldehyde dehydrogenase have previously been isolated and characterized from some mesophilic and thermophilic (but not hyper- or extremely thermophilic) bacteria, including members of the genera* Thermoanaerobacter* and* Geobacillus*, with optimal temperatures of approximately 55–60°C [22, 23]. The AcDH (CoA-acetylating) encoding genes were absent from the released genome sequences of hyperthermophiles. CFEs of different hyperthermophilic bacteria (*T. maritima* and* T. hypogea*) and archaea (*P. furiosus*,* T. kodakaraensis*, and* T. guaymasensis*) indeed lacked detectable CoA-dependent AcDH activity when they are examined under a variety of assay conditions (data not shown).

PDC activity catalyzed by the bifunctional TgPOR/PDC that was characterized here can complete our understanding of ethanol production pathways in* Thermococcales.* Considering the very high activity of the zinc-containing ADH characterized from* T. guaymasensis* (223 U mg^−1^ in the aldehyde/ketone reduction direction) and relatively low PDC activity of the enzyme characterized here (3.8 ± 0.22 U mg^−1^), it appears that PDC activity is the limiting factor and the reason for low ethanol yield of hyperthermophiles. The PDC specific activities of the purified bifunctional PORs/PDCs are much lower than the activities of the PDCs from yeasts and bacteria. For instance, the PDCs from* Zymobacter palmae, Sarcina ventriculi, Z. mobilis, S. cerevisiae,* and* Zea mays* have the specific activities of 66, 103, 160, 40, and 96 U mg^−1^, respectively [16, 17, 50, 56, 57]. In hyperthermophiles, pyruvate is most likely catabolized by POR reaction, leading to the production of acetyl-CoA, which is either used for biosynthesis purposes or is converted to acetate by acetyl-CoA synthetase due to the absence of CoA-dependent AcDH activity [58]. The production of acetate is coupled to energy conservation in the form of ATP (Figure 6).

For more than a decade, PfPOR has been considered to be the sole acetaldehyde-producing enzyme known in hyperthermophiles. Although production of acetaldehyde was shown by POR/PDC* in vitro* and the enzymes are isolated from ethanologenic microorganisms, it is still not known that what factors and/or physiological conditions favor one activity over another.* In vivo* studies such as microarray and real-time PCR combined with enzyme activity studies may be exploited to determine the physiological conditions and substrates that regulate each of the activities. Also, detailed analysis of the end products under each growth condition may further clarify potential roles of each enzyme activity. The elucidation of the catalytic mechanism will be helpful in determining the amino acid residues that might be involved in nonoxidative decarboxylation of pyruvate (PDC activity) catalyzed by the bifunctional enzymes.

## 5. Conclusions

In this study, it was shown that pyruvate ferredoxin oxidoreductases from the hyperthermophilic archaeon* T. guaymasensis* and most likely all *β*-keto acid ferredoxin oxidoreductases (KORs) can catalyze the nonoxidative decarboxylation of keto acids to produce corresponding aldehydes [6, 9]. Hence, KORs may be regarded as regulating factors for conversion of keto acids to both acyl-CoA and corresponding aldehyde. The bifunctional PDC/POR is mainly different from the commonly known PDC in terms of CoA-dependency, oxygen sensitivity, and lower catalytic activity, representing a novel type of PDCs. A better understanding of the catalytic mechanism and metabolic regulation of the bifunctional PDC/POR requires further investigation.

## Figures and Tables

**Table 1:** Purification of bifunctional PDC/POR from *T. guaymasensis* ^a^.

Step	Total protein^b^ (mg)	Total activity^c^ (units)	Specific activity^d^ (U mg^−1^)	Purification (fold)	Recovery (%)
Cell-free extract	1358	3434	2.6	1	100
DEAE Sepharose	322	1748	5.6	2.2	50.9
Hydroxyapatite	84	580	7.5	2.9	16.9
Phenyl Sepharose	17.9	363	20.2 ± 1.8	7.8	10.6

^a^CFE was prepared from 25 g (wet weight) of *T. guaymasensis *cells.

^b^As determined using Bradford assay as described in the Materials and Methods.

^c^The POR activity assayed as described in the material and methods.

^d^One unit was defined as one *μ*mol of pyruvate oxidized min^−1^.

**Table 2:** Purification of bifunctional PDC/POR from *P. furiosus* ^a^.

Step	Total protein^b^ (mg)	Total activity^c^ (units)	Specific activity^d^ (U mg^−1^)	Purification (fold)	Recovery (%)
Cell-free extract	1267	8442	3.2	1	100
DEAE Sepharose	386	2016	5.2	1.7	23.8
Phenyl Sepharose	80	1031	13.1	4.1	12.2
Gel filtration	28.2	495	19.1	6.0	5.8
Hydroxyapatite	21	392	22.3 ± 2.2	7.0	4.6

^a^CFE was prepared from 20 g (wet weight) of *P. furiosus *cells.

^b^As determined using Bradford assay as described in the Materials and Methods.

^c^POR activity assayed as described in the Material and Methods.

^d^One unit was defined as one *μ*mol of pyruvate oxidized min^−1^.

**Table 3:** Kinetic parameters of POR and PDC of *T. guaymasensis* and *P. furiosus*.

Source	activity	Pyruvate^a^		CoA^b^		Specific activity^c^
		Apparent *K*_*m*_ (mM)	Apparent *V*_max⁡_ (U mg^−1^)	Apparent *K*_*m*_ (*μ*M)	Apparent*V*_max⁡_ (U mg^−1^)	
*T. guaymasensis *	POR	0.53 ± 0.03	18 ± 0.23	70 ± 10	21.8 ± 0.8	20.2 ± 1.8
	PDC	0.25 ± 0.05	3.8 ± 0.14	20 ± 1	3.3 ± 0.09	3.8 ± 0.22

*P. furiosus *	POR^d^	0.46	23.6	110	39.9	22.0
	PDC^e^	1.1	4.3 ± 0.3	110	4.3 ± 0.3	4.3 ± 0.3

^a^For POR measured at 0.1 mM CoA, 1 mM MV, 0.4 mM TPP, and for PDC at 1 mM CoA, 0.1 mM TPP.

^b^For POR measured at 5 mM pyruvate, 1 mM MV, and 0.4 mM TPP, and for PDC at 10 mM pyruvate and 0.1 Mm TPP.

^c^Expressed as *μ*moles of pyruvate oxidized^−1^ mg^−1^ of enzyme.

^d^Values reported by Blamey and Adams (1993) [28] measured using EPPS (50 mM, pH 8.4) at 80°C.

^e^Values reported by Ma et al. (1997) [6] measured using CAPS (50 mM, pH 10.2) at 80°C.
